# Cross-lagged analysis of depression symptoms and internet addiction tendencies among first-year university students and the moderating effects of urban–rural background and sibling status

**DOI:** 10.3389/fpsyt.2026.1916116

**Published:** 2026-08-26

**Authors:** YuWei Yang, YiPeng Su, GuoGuang Cao, Bo Liu, XinFeng Zhang, XinWei Yu, Li Zhang, Ye Yu, XueJian Su, DeFa Mo, LiFang Zhou, XiaoPeng Deng

**Affiliations:** 1 Jingzhou Hospital Affiliated to Yangtze University, Jingzhou, Hubei, China; 2 Mental Health Institute of Yangtze University, Jingzhou, Hubei, China; 3 Jingzhou Mental Health Center, Jingzhou, Hubei, China; 4 Center for Student Mental Health Education, Yangtze University, Jingzhou, Hubei, China; 5 Jingzhou Rongjun Special Care Hospital, Jingzhou, Hubei, China

**Keywords:** cross-lagged analysis, depression symptoms, internet addiction, only-child status, university students, urban-rural background

## Abstract

**Objective:**

To reveal the reciprocal predictive relationship between depressive symptoms (PHQ-9) and internet addiction tendencies (IAT) among first-year university students, and to compare differences across urban-rural backgrounds and only-child status, providing a basis for targeted interventions.

**Methods:**

This study conducted follow-up surveys of 6, 070 freshmen of the 2023 cohort at a university in Jingzhou, Hubei Province, in October 2023 and May 2024. Controlling for gender, household economic status, and family structure, cross-lagged path analysis was used to examine the dynamic association between depression symptoms and internet addiction tendencies.

**Results:**

T1 and T2 PHQ-9 and IAT scores were significantly positively correlated (T1: r=0.453; T2: r=0.485, p<0.01). Nonparametric tests showed significant urban–rural differences for T1 PHQ-9 (Z = 3.069, p=0.002), T2 PHQ-9 (Z = 4.329, p<0.001), T1 IAT (Z = 5.079, p<0.001), and T2 IAT (Z = 5.335, p<0.001); significant only-child differences for T1 PHQ- (Z = 4.173, p<0.001), T2 PHQ-9 (Z = 4.980, p<0.001), T1 IAT (Z = 7.189, p<0.001), and T2 IAT (Z = 6.040, p<0.001). Cross-lagged analyses of the full model indicated bidirectional prediction. In subgroup models, T1 IAT significantly predicted T2 PHQ-9 in all groups: urban only-child (β=0.172, p<0.001), urban non-only-child (β=0.173, p<0.001), rural only-child (β=0.233, p<0.001), and rural non-only-child (β=0.238, p<0.001). T1 PHQ-9 significantly predicted T2 IAT only in urban only-child (β=0.109, p<0.001) and rural non-only-child (β=0.099, p<0.001), but not in urban non-only-child or rural only-child.

**Conclusions:**

Internet addiction tendency is significantly correlated with depressive symptoms. At the group level, the baseline level of internet addiction tendency positively predicts subsequent changes in depressive symptoms, and this effect holds true across all groups. The predictive effect of depressive symptoms on subsequent internet addiction tendency is significant only in the urban only-child and rural non-only-child groups in subgroup analyses. However, further multi-group comparisons show that the between-group difference in this path is not statistically significant, so a definitive group moderation effect cannot yet be inferred.

## 1 Introduction

Internet addiction (IA) refers to a condition in which excessive use of the internet leads to impaired functioning and causes damage at cognitive, psychological, or physical levels (1). In today’s global digital era, the internet has become the main means for people to socialize and entertain online. However, the rapid development of the internet has also brought problems related to pathological overuse; long-term and uncontrolled internet use can evolve into internet addiction tendency. Another impact of COVID-19 pandemic and its consequences is that people in the world tend to change their lifestyle and youth, adolescents in particular: from restricting physical activities and open spaces during high school, they are also more dependent on the Internet after entering college, further aggravating the risk of internet addiction tendency in college students. At the same time, continued internet use frequency in college students has brought increasingly serious mental problems, negative psychological consequences are becoming increasingly evident (2).

College students are a special group in a pivotal transition from adolescence to adulthood. They are in the life stages of stress in the most critical period of their lifetime (3). Earlier research shows that the prevalence of depression symptoms among university students in most parts of the world is always rising and more than 2/3 young people are silent and reluctant to talk or ask for help about mental health problems (4). Because of this introverted coping style, college students are easy to become addicted to the Internet and meanwhile likely to develop internet addiction tendency (5).

In recent years, because of the popularity of smartphones and the internet, occurrence of internet addiction tendency gradually appears and has a relatively high comorbidity with psychological problems such as depression symptoms and anxiety (6, 7). Ramón-Arbués and colleagues’survey of 1, 074 Spanish university students found that problematic internet use is a significant risk factor for psychological distress such as depressive symptoms, anxiety, and stress (7). Similarly, more studies have confirmed a significant association between internet addiction tendency and mental health problems (8, 9). A cross-sectional survey involving 2, 050 adolescents suggested that depressive symptoms are a potential factor influencing internet addiction tendency (8). Studies by Yang X (10), Zhao Y (11), and others also support the high comorbidity between internet addiction tendency and depressive symptoms. Interestingly, in a wider population, a nationwide online study of 4734 adults in Indonesia reported a positive association of internet addiction tendency with depressive symptoms (12).In summary, depressive symptoms are a risk factor for internet addiction tendency, often coexist and produce a vicious circle whereby the 2 conditions reinforce each other.

Against a backdrop of decades of the one-child policy, only children and non-only children can be different in upbringing, family resources and psychological development. Whether these differences influence internet addiction tendency and mental health has attracted rich research attention. Early studies refuted stereotypes such as ‘only children are more sensitive’: Chinese children with siblings exhibited higher fear, anxiety and depressive symptoms on indices (13), and only children did not differ to non-only children in the important measures such as suicidal tendencies (14). Another study indicated a slight psychological health benefit of only children, stronger among those born after one-child policy implementation (15). Other than that, “only-child advantage”may derive from”concentrated family resource investment, “which makes only-child status a protective factor, but it also reflects that the advantage of being an only child is closely related to the social acceptance environment of an individual (16). A study of Chinese adults found that the people without siblings also had better subjective mental health, with the”only-child advantage”being more obvious for the rural residents and the women (17). This suggests that the urban-rural context and only-childhood status may interact in influencing individual mental health. But based on the crude categories of”only child or not only child”will render the important role played by the quality of intra-family relationship and intra-family support pattern hidden.A recent experiment of the social buffering role of siblings was the first to consider physiological causes and found that the buffering role of siblings did exist among adolescents with high depressive symptoms, and that the buffering role had strong effect of relationship quality, especially feeling inalienable in a relationship (18).

In summary, prior longitudinal and cross-sectional studies provide us a general direction to understand how sibling status and urban–rural factors affect the mental health of individual. How these factors moderate the bidirectional relationships between depressive symptoms and internet addiction tendency across different groups, however, remains unknown. Building on the above, this study adopted the cross-lagged method to explore the interactive influence of urban–rural differences and sibling status on the longitudinal relationship between two, which provides the basis for the following formulation of differentiated intervention strategies.Recent evidence suggests that emotional dysregulation may be a core mechanism through which depressive symptoms and addictive behaviors, including internet addiction, reinforce each other. When negative emotions are not adequately counterbalanced, individuals—particularly during the transition from adolescence to young adulthood—may resort to problematic internet use as a maladaptive coping strategy, which in turn deepens depressive affect and creates a vicious cycle. Sibling status and urban–rural background, as factors that shape emotional socialization and family support resources, are likely to moderate this cycle. The present study therefore tests whether these two contextual variables moderate the longitudinal bidirectional relationship between depressive symptoms and internet addiction tendency, offering a more nuanced perspective for targeted prevention (19).

## 2 Subjects and methods

### 2.1 Study subjects

In October 2023 (T1), a total of 8498 first-year students from comprehensive universities were recruited using cluster sampling to participate in this survey. In May 2024 (T2), questionnaires were distributed to all students, and 8672 students participated. After removing participants with duplicate responses, missing key variables such as name, or response times that were too short (less than 2 seconds per item) (20), the valid number of participants at T1 was 7353. After follow-up matching, a total of 6070 students completed the questionnaire at both time points. This study was approved by the Ethics Committee of Jingzhou Mental Health Center (Ethics Approval Numbers: 2023LL0808 and 2024LL0801). Prior to signing informed consent forms, all participants were informed about the study’s purpose, procedures, privacy protection, risks, and data retention.

### 2.2 Research instruments

#### 2.2.1 General demographic questionnaire

The study collected information including age, gender, family structure, place of origin, and whether participants have siblings. The sample consisted of 1, 283 only children in urban areas, 1, 502 non-only children in urban areas, 651 only children in rural areas, and 2, 634 non-only children in rural areas.

#### 2.2.2 Internet addiction test

The Internet Addiction Test (IAT) compiled by Young (1996) was used to assess participants’ tendency toward internet addiction (21). The scale contains 20 items and uses a five-point scoring system (1 = Almost never, 2 = Occasionally, 3 = Sometimes, 4 = Often, 5 = Always), with a total score range of 20–100; higher scores indicate a more severe tendency toward internet addiction (22). Previous systematic reviews have shown that the IAT demonstrates good internal consistency across different samples (Cronbach’s α typically ranging from 0.85 to 0.95) (22); a validation study among Chinese undergraduates also reported a Cronbach’s α of 0.932 for the scale (23). Based on prior research standards, a total score<50 is defined as normal internet use, whereas ≥50 is defined as indicating a tendency toward internet addiction (24, 25). In this study, the Cronbach’s α for IAT scores was 0.886 at T1 and 0.901 at T2; acceptable Cronbach’s α values range from 0.70 to 0.95 (26).

#### 2.2.3 Patient health questionnaire-9

The Patient Health Questionnaire-9 (PHQ-9), developed by Kroenke et al. in 2001 and validated in Chinese by Wang et al. in 2014, was used to assess the severity of participants’ depressive symptoms over the past two weeks. The scale’s Cronbach’s α in Chinese community populations is 0.86 (27). The scale consists of 9 items corresponding to the nine core depressive symptoms in DSM-5. Each item has four response options on a four-point scale: 0 = Not at all (0 days), 1 = Several days (1–6 days), 2 = More than half the days (7–13 days), 3 = Nearly every day (14 days or more); total scores range from 0 to 27, with higher scores indicating more severe depressive symptoms (28). In this study, the Cronbach’s α for PHQ-9 scores was 0.869 at T1 and 0.929 at T2.

### 2.3 Statistical analysis

Statistical analyses were performed using SPSS (version 27) and RStudio (version 4.5.1). The final analysis sample included only participants who completed both surveys, and there were no missing values in the data; therefore, no other missing data handling methods were used. First, the data were cleaned and organized, and nonparametric tests were used to examine associations between each variable and the dependent variable. Second, descriptive statistics were used to analyze the distribution characteristics of the four groups on variables such as sex, only-child status, internet addiction tendency, and depressive symptoms. Third, correlation analyses were conducted to examine relationships among variables both with and without controlling for covariates. Fourth, prior to constructing the cross-lagged model, measurement invariance of the PHQ-9 and IAT scales across the four groups (urban only-child, urban non-only-child, rural only-child, and rural non-only-child) was examined using multi-group confirmatory factor analysis. The primary criteria for establishing invariance were ΔCFI≥-0.010 and ΔRMSEA ≤ 0.015 (29); given the large sample size, the robust chi-square difference test was used only as a supplementary reference. After measurement invariance was confirmed, multicollinearity among the variables was assessed using the variance inflation factor (VIF) and tolerance. Finally, a longitudinal cross-lagged model was constructed. Model fit was evaluated comprehensively by the comparative fit index (CFI), Tucker-Lewis Index (TLI), root mean square error of approximation (RMSEA), and standardized root mean square residual (SRMR): CFI > 0.90, TLI > 0.90, RMSEA< 0.08, and SRMR< 0.08 indicate good model fit (30). The significance levels in this study were P< 0.05, P< 0.01, and P< 0.001. To test for between-group differences in the cross-lagged paths, Wald chi-square tests were used to formally compare the two key paths, “T1 internet addiction tendency → T2 depressive symptoms” and “T1 depressive symptoms → T2 internet addiction tendency, “ to assess whether the between-group differences were statistically significant.

## 3 Results

### 3.1 Common method bias test

To test for common method bias, Harman’s single-factor test was performed separately on the PHQ-9 and IAT scale data at T1 and T2. The results showed that the first factor of the PHQ-9 at T1 explained 49.05%, below the empirical threshold of 50%, indicating no serious common method bias (31). At T2 the first factor explained 55.49%, above the 50% threshold, so confirmatory factor analysis (CFA) was further conducted on the T2 data (32). The two-factor revised model fit was: χ² (25)=2355.60, CFI = 0.934, TLI = 0.908, RMSEA = 0.102, SRMR = 0.043; the single-factor model fit was: χ²(27)=2725.72, CFI = 0.927, TLI = 0.903, RMSEA = 0.107, SRMR = 0.046. The two-factor revised model was superior to the single-factor model (Δχ²(2)=370.12, p<0.001). which provides some relative evidence for distinguishing trait and method effects. However, the RMSEA of the two-factor model remained above the commonly used cut-off value of 0.08, suggesting poor absolute model fit and indicating that common method variance may still be a potential factor affecting the data structure. For the IAT, the first factor explained 32.76% at T1 and 35.73% at T2, both below the 50% empirical threshold, indicating no serious common method bias in either case.

In summary, no extreme situation in which a single factor explained the vast majority of variance was observed in the present data. However, given that all variables were derived from participants’ self-reports, the influence of shared method variance on the observations cannot be completely ruled out.

### 3.2 Measurement invariance testing

To examine whether the IAT and PHQ-9 have measurement equivalence across groups defined by urban/rural background and sibling status, multi-group confirmatory factor analysis was conducted separately on the T1 and T2 data. (1) For the IAT at T1, the configural invariance model showed acceptable fit (CFI = 0.856, RMSEA = 0.068, SRMR = 0.047). Metric invariance was supported (ΔCFI=−0.0004, ΔRMSEA=−0.0026; Δχ²(57)=52.36, p=0.650). Scalar invariance was also supported (ΔCFI=−0.0018, ΔRMSEA=−0.0020); although the robust chi-square difference test was significant (Δχ²(57)=117.75, p<0.001), given the large sample size, ΔCFI and ΔRMSEA were still used as the primary criteria (33). For the IAT at T2, the configural model showed acceptable fit (CFI = 0.852, RMSEA = 0.076, SRMR = 0.051); both metric invariance (ΔCFI=−0.0011, ΔRMSEA=−0.0027; Δχ²(57)=108.87, p<0.001) and scalar invariance (ΔCFI=−0.0025, ΔRMSEA=−0.0021; Δχ²(57)=158.87, p<0.001) were within the acceptable range.(2) For the PHQ-9 at T1, the configural invariance model showed acceptable fit (CFI = 0.948, RMSEA = 0.079, SRMR = 0.034). Both metric invariance (ΔCFI=−0.0006, ΔRMSEA=−0.0071; Δχ²(24)=18.21, p=0.793) and scalar invariance (ΔCFI=−0.0003, ΔRMSEA=−0.0056; Δχ²(24)=30.06, p=0.183) were within the acceptable range. For the PHQ-9 at T2, the configural model’s CFI and SRMR were acceptable (CFI = 0.921, SRMR = 0.044), but the RMSEA was slightly high (0.110), which may be attributable to the estimation properties of RMSEA when the scale has few items and the model has low degrees of freedom (34). Metric invariance (ΔCFI=−0.0015, ΔRMSEA=−0.0096; Δχ²(24)=28.59, p=0.236) and scalar invariance (ΔCFI=−0.0005, ΔRMSEA=−0.0078; Δχ²(24)=37.65, p=0.038) were both within the acceptable range. Although the robust chi-square difference test for the scalar model was marginally significant (p=0.038), given the large sample size, ΔCFI and ΔRMSEA were still used as the primary criteria.

Overall, the IAT and PHQ-9 scales achieved scalar invariance at both T1 and T2, allowing for subsequent cross-group comparisons.

### 3.3 Sensitivity analysis

Attrition analysis showed that the dropout sample (n=1283) and the final analytic sample (n=6070) did not differ significantly on baseline family structure (χ²=0.179, P = 0.673), household economic status (χ²=1.711, P = 0.425), or depressive symptoms (Z≈0.000, P = 0.821). However, differences were statistically significant for urban–rural status (χ²=5.086, P = 0.024), sex (χ²=82.423, P<0.001), only-child status (χ²=7.686, P = 0.006), and tendency toward internet addiction (Z = 2.165, P = 0.030). Given the large sample size (total effective sample N = 6070), effect sizes were further calculated to assess the practical significance of these differences. For categorical variables, the Phi coefficient for urban-rural status was φ=0.026, for sex wasφ=0.106, and for only-child status was φ=0.032. For the continuous variable, the effect size for internet addiction tendency was r=0.025. All were small effects.

### 3.4 Demographic characteristics

At T1 in the total sample, age had a mean and standard deviation of M = 18.91 ± SD = 2.12. There were 2872 males (47.31%) and 3198 females (52.69%). Regarding household economic status, 1490 participants (24.55%) reported poorer economic conditions, 4431 (73.00%) reported average, and 149 (2.46%) reported better conditions. In terms of family structure, 5497 participants (90.56%) came from intact families and 573 (9.44%) from non-intact families. After cross-classifying urban–rural status with only-child status, at T1 the PHQ-9 scores M(P25, P75) were: urban only-child group 3.0 (1.0, 6.0), urban non-only-child group 3.0 (1.0, 6.0), rural only-child group 3.0 (1.0, 6.0), rural non-only-child group 4.0 (1.0, 6.0); the IAT scores M(P25, P75) were: urban only-child group 32.0 (25.0, 40.0), urban non-only-child group 35.0 (28.0, 42.0), rural only-child group 34.0 (27.0, 41.0), rural non-only-child group 35.0 (28.0, 42.0). At T2 the PHQ-9 scores M(P25, P75) were 2.0 (0.0, 4.0) for all four groups; the IAT scores M(P25, P75) were: urban only-child 32.0 (25.0, 40.0), urban non-only-child group 34.0 (26.0, 41.0), rural only-child group 34.0 (26.0, 41.0), rural non-only-child group 35.0 (27.0, 43.0).

To examine differences in depressive symptoms (PHQ-9, T1/T2) and Internet addiction tendency (IAT, T1/T2) across demographic characteristics, Mann–Whitney U tests (urban/rural, only child status, gender, family structure) and Kruskal–Wallis H tests (Household economic status) were used. Results showed: Urban/rural differences were significant for PHQ-9 scores at T1 (Z = 3.069, p=0.002, r=0.039), PHQ-9 scores at T2 (Z = 4.329, p<0.001, r=0.056), IAT scores at T1 (Z = 5.079, p<0.001, r=0.065), and IAT scores at T2 (Z = 5.335, p<0.001, r=0.068), with urban participants scoring lower than rural participants. Only-child status was significant for PHQ-9 at T1 (Z = 4.173, p<0.001, r=0.054), PHQ-9 at T2 (Z = 4.980, p<0.001, r=0.064), IAT at T1 (Z = 7.189, p<0.001, r=0.092), and IAT at T2 (Z = 6.040, p<0.001, r=0.078), with only children scoring lower than non-only children. Sex was significant only for IAT at T1 (Z = 3.832, p<0.001, r=0.049), with males scoring higher than females; all other indicators were not significant: PHQ-9 at T1 (Z = 1.368, p=0.171), PHQ-9 at T2 (Z = 1.882, p=0.060), and IAT at T2 (Z = 1.685, p=0.092). Family structure was only marginally significant for PHQ-9 at T1 (Z = 1.960, p=0.050, r=0.025), with participants from intact families scoring slightly lower than those from non-intact families; other indicators were not significant: PHQ-9 at T2 (Z = 0.341, p=0.733), IAT at T1 (Z = 0.415, p=0.678), and IAT at T2 (Z = 1.365, p=0.172). Family economic status was significant across all four indicators: PHQ-9 at T1 (H = 38.554, p<0.001, η²=0.006), PHQ-9 at T2 (H = 25.196, p<0.001, η²=0.004), IAT at T1 (H = 16.803, p<0.001, η²=0.003), and IAT at T2 (H = 19.268, p<0.001, η²=0.003). All the above effect sizes were small, suggesting that the actual magnitude of the between-group mean differences was limited in the context of a large sample. See **Table 1** for details.

**Table 1:** Differences in depressive symptoms and internet addiction tendency across different demographic characteristics.

Variable	PHQ-9 score M (P25, P75)		IAT Scores M (P25, P75)	
	T1	T2	T1	T2
Urban-Rural Differences				
Urban (N=2785)	3.0 (1.0, 6.0)	2.0 (0, 5.0)	34.0 (27.0, 41.0)	33.0 (25.0, 40.0)
Rural (N=3285)	3.0 (1.0, 6.0)	2.0 (0, 5.0)	35.0 (28.0, 43.0)	35.0 (27.0, 42.0)
Z/P value	3.069/0.002	4.329/<0.001	5.079/<0.001	5.335/<0.001
Sibling status				
Only child (N=1934)	3.0 (1.0, 6.0)	2.0 (0, 5.0)	33.0 (26.0, 41.0)	32.0 (25.0, 40.0)
Not an only child (N=4136)	3.0 (1.0, 6.0)	2.0 (0, 5.0)	35.0 (28.0, 43.0)	35.0 (27.0, 42.0)
Z/P value	4.173/<0.001	4.980/<0.001	7.189/<0.001	6.040/<0.001
Gender				
Male (N=2872)	3.0 (1.0, 6.0)	2.0 (0, 5.0)	34.0 (27.0, 42.0)	34.0 (26.0, 41.0)
Female (N=3198)	3.0 (1.0, 6.0)	2.0 (0, 5.0)	35.0 (28, 43.0)	34.0 (27.0, 42.0)
Z/P value	1.368/0.171	1.882/0.060	3.832/<0.001	1.685/0.092
Family structure				
Intact family (N=5497)	3.0 (1.0, 6.0)	2.0 (0, 5.0)	35.0 (27, 42.0)	34.0 (26.0, 41.0)
Non-intact family (N=573)	4.0 (1.0, 7.0)	2.0 (0, 5.0)	35.0 (27.0, 42.0)	33.0 (25.5, 41.0)
Z/P value	1.960/0.050	0.341/0.733	0.415/0.678	1.365/0.172
Household economic status				
Poor (N=1490)	4.0 (1.0, 7.0)	3.0 (0, 6.0)	36.0 (28.8, 43.3)	35.0 (27.0, 43.0)
General (N=4431)	3.0 (1.0, 6.0)	2.0 (0, 5.0)	34.0 (27.0, 42.0)	33.0 (26.0, 41.0)
Better (N=149)	2.0 (0, 5.5)	2.0 (0, 4.0)	34.0 (27.0, 39.5)	31.0 (25.0, 40.0)
H/P value	38.554/<0.001	25.196/<0.001	16.803/<0.001	19.268/<0.001

### 3.5 Correlation analysis

According to the correlation matrix results, the variables were significantly positively correlated (p<0.01). T1 depressive symptoms were correlated with T2 depressive symptoms (r=0.411), and T1 Internet addiction tendency was highly correlated with T2 Internet addiction tendency (r=0.513); cross-time correlations were also significant: T1 depressive symptoms with T2 Internet addiction tendency (r= 0.300) and T1 Internet addiction tendency with T2 depressive symptoms (r=0.327) were both moderately correlated, suggesting a possible bidirectional relationship between the two. See **Table 2** for details.

**Table 2:** Correlation matrix among the main variables of the study.

#	Variables	1	2	3	4
1	T1Depressive symptoms	—	0.411^**^	0.453^**^	0.300^**^
2	T2Depressive symptoms	0.411**	—	0.327^**^	0.485^**^
3	T1Internet addiction tendency	0.452**	0.327**	—	0.513^**^
4	T2Internet addiction tendency	0.299**	0.485**	0.513**	—

(1) The lower-left indicates the correlation coefficient without controlling covariates (gender, family structure, Household economic status); the upper-right indicates the correlation coefficient after controlling covariates. (2) **indicates p<0.01.

### 3.6 Cross-lagged model

To assess the degree of multicollinearity among predictors in the regression models, we calculated variance inflation factors (VIF) and tolerance. A VIF > 4.0 or tolerance< 0.25 indicates a collinearity issue that warrants attention (35). After adjustment, the T1 PHQ-9 score (baseline depressive symptoms) had a VIF of 1.18 and tolerance of 0.845; the T2 PHQ-9 score (follow-up depressive symptoms) had a VIF of 1.21 and tolerance of 0.828; the T1 IAT score (baseline tendency toward Internet addiction) had a VIF of 1.25 and tolerance of 0.797; the T2 IAT score (follow-up tendency toward Internet addiction) had a VIF of 1.27 and tolerance of 0.788. Therefore, the risk of multicollinearity in the study was low, and on this basis we proceeded to build a cross-lagged model to examine the longitudinal relationships among the variables.

**Figure 1:**
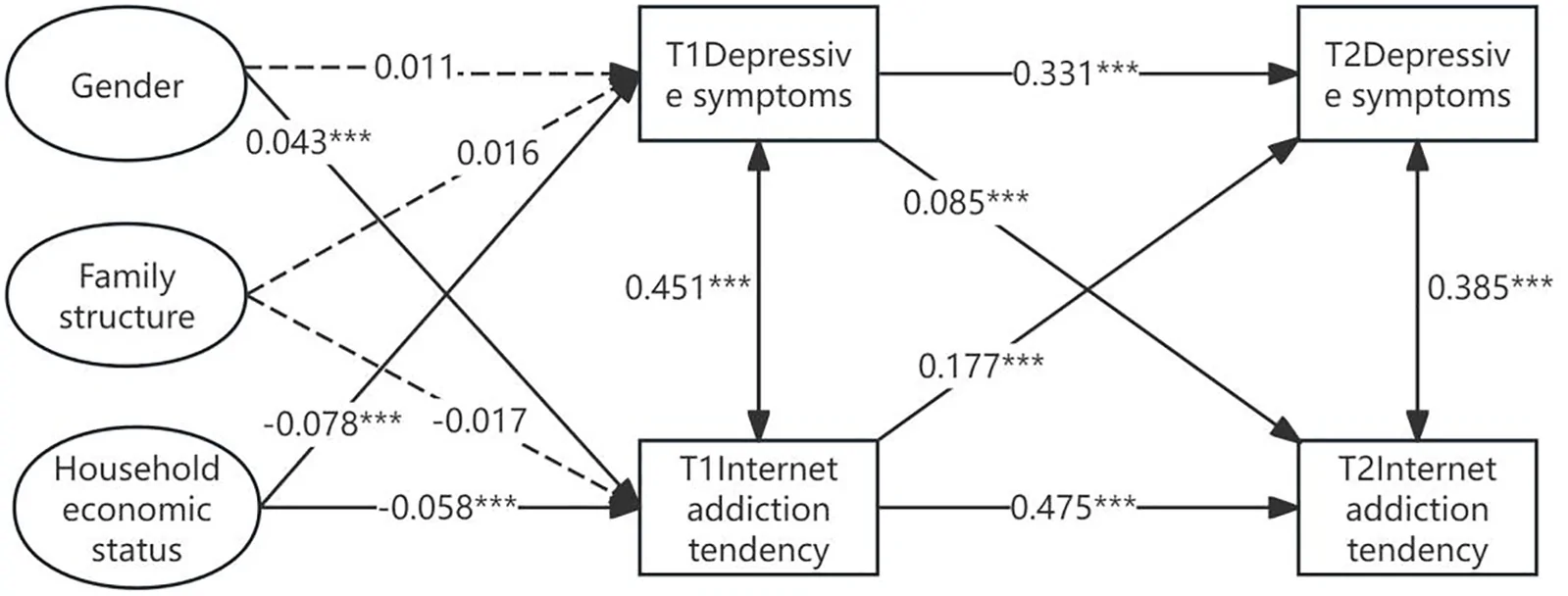
Overall model after controlling for covariates. Note: Solid lines in the path indicate p < 0.05, dashed lines indicate p > 0.05. Numbers on the lines represent standardized β coefficients. * p < 0.05, ** p < 0.01, *** p < 0.001.

After controlling for covariates (gender, family structure, and Household economic status), the overall cross-lagged model showed good fit:χ²(6)=11.1, p=0.086, CFI = 0.999, TLI = 0.997, RMSEA = 0.012, SRMR = 0.007. A bidirectional cross-lagged effect was observed between depressive symptoms and internet addiction tendency. The R² for T2 depressive symptoms was 0.194, and the R² for T2 internet addiction tendency was 0.269. The unstandardized coefficients (B), standard errors (SE), standardized coefficients (β), and 95% confidence intervals for the main paths were as follows: T1 internet addiction tendency positively predicted T2 depressive symptoms (B = 0.064, SE = 0.005, β=0.180, 95%CI[0.055, 0.073], p<0.001), and T1 depressive symptoms positively predicted T2 internet addiction tendency (B = 0.226, SE = 0.033, β=0.09, 95%CI[0.162, 0.290], p<0.001). household economic status had a weak negative predictive effect on T1 depressive symptoms (β=−0.078, p<0.001) and T1 internet addiction tendency (β=−0.058, p<0.001); sex had a weak positive predictive effect only on T1 internet addiction tendency (β=0.043, p=0.001); family structure did not significantly predict T1 depressive symptoms or T1 internet addiction tendency (p>0.05), as shown in **Figure 1**.

**Figure 2:**
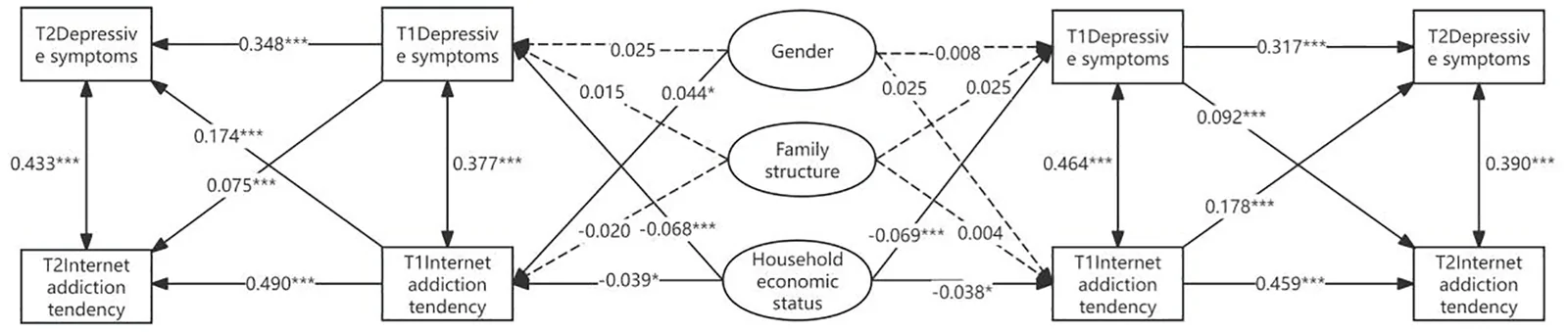
Cross-lagged model after grouping by urban-rural differences. Note: The left side is the urban group, and the right side is the rural group. Solid lines in the path indicate p < 0.05, dashed lines indicate p > 0.05. Numbers on the lines represent standardized β coefficients. * p < 0.05, ** p < 0.01, *** p < 0.001.

Using urban–rural differences as a grouping variable, a multi-group cross-lagged analysis was conducted. Both the urban and rural group models showed good fit (urban group: χ²(8)=8.342, CFI = 1.000, TLI = 1.000, RMSEA = 0.004, SRMR = 0.009; rural group: χ²(8)=12.193, CFI = 0.999, TLI = 0.996, RMSEA = 0.013, SRMR = 0.010). The R²for T2 depressive symptoms was 0.203 and for T2 internet addiction tendency was 0.278 in the urban group; the R²for T2 depressive symptoms was 0.185 and for T2 internet addiction tendency was 0.258 in the rural group. The unstandardized coefficients (B), standard errors (SE), standardized coefficients (β), and 95% confidence intervals for the main paths were as follows: T1 internet addiction tendency positively predicted T2 depressive symptoms (urban group:B=0.062, SE = 0.008, β=0.174, 95% CI [0.046, 0.078], p<0.001; rural group: B = 0.065, SE = 0.007, β=0.178, 95%CI[0.051, 0.080], p<0.001), and T1 depressive symptoms positively predicted T2 internet addiction tendency (urban group: B = 0.203, SE = 0.056, β=0.075, 95%CI[0.093, 0.313], p<0.001; rural group:B=0.243, SE = 0.053, β=0.092, 95% CI [0.138, 0.348], p<0.001). See **Figure 2** for details.

To further examine the interaction between urban-rural background and only-child status, urban-rural differences and only-child status were crossed to form groups, and corresponding cross-lagged models were constructed. Both the urban only-child group and the urban non-only-child group showed good fit (urban only-child group fit: χ²(6)=6.49, p=0.370, CFI = 1.000, TLI = 0.999, RMSEA = 0.008, SRMR = 0.010;urban-non-only-child group: χ²(6)=10.30, CFI = 0.997, TLI = 0.991, RMSEA = 0.022, SRMR = 0.016).

In the urban only-child group, the R² for T2 depressive symptoms was 0.200 and for T2 internet addiction tendency was 0.287. The unstandardized coefficients (B), standard errors (SE), standardized coefficients (β), and 95% confidence intervals for the main paths were as follows: T1 internet addiction tendency positively predicted T2 depressive symptoms (B = 0.063, SE = 0.012, β=0.172, 95% CI [0.040, 0.086], p<0.001), and T1 depressive symptoms also positively predicted T2 internet addiction tendency (B = 0.292, SE = 0.087, β=0.109, 95%CI[0.123, 0.462], p=0.001). In the urban non-only-child group, the R² for T2 depressive symptoms was 0.205 and for T2 internet addiction tendency was 0.268. The unstandardized coefficients (B), standard errors (SE), standardized coefficients (β), and 95% confidence intervals for the main paths were as follows: T1 internet addiction tendency positively predicted T2 depressive symptoms (B = 0.061, SE = 0.011, β=0.173, 95% CI [0.039, 0.083], p<0.001), but the prediction from T1 depressive symptoms to T2 internet addiction tendency was not significant (B = 0.124, SE = 0.073, β=0.046, 95% CI [−0.020, 0.267], p=0.092).

**Figure 3:**
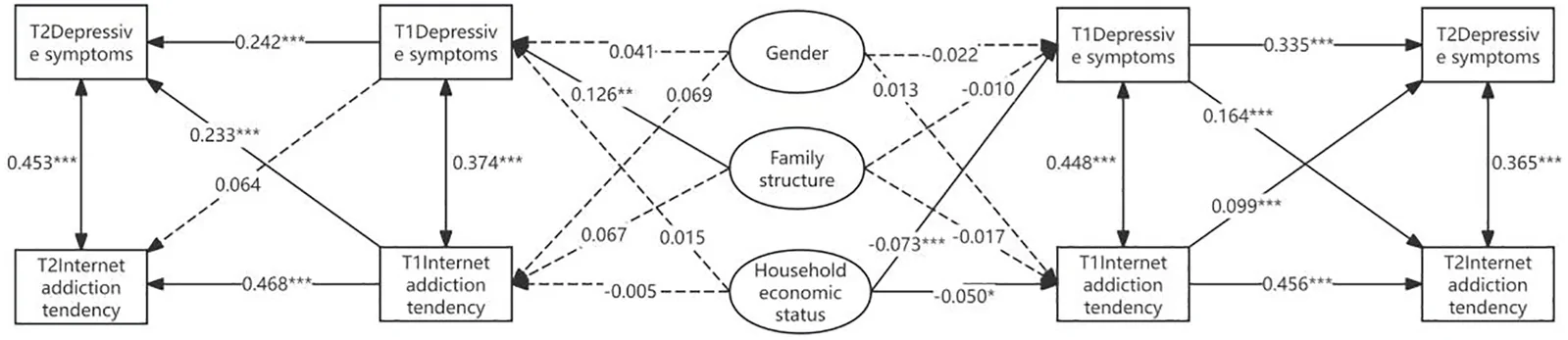
Cross-lagged model after cross-grouping. Note: The left side is the urban only-child group, and the right side is the urban non-only-child group. Solid lines in the path indicate p < 0.05, dashed lines indicate p > 0.05. Numbers on the lines represent standardized β coefficients. * p < 0.05, ** p < 0.01, *** p < 0.001.

In the cross-lagged paths, T1 tendency toward Internet addiction predicted T2 depressive symptoms significantly in both the only-child and non-only-child groups. However, T1 depressive symptoms predicted T2 tendency toward Internet addiction only in the urban only-child group and not significantly in the urban non-only-child group. The autoregressive paths for depressive symptoms and tendency toward Internet addiction were significant in both groups. See **Figure 3** for details.

A cross-lagged model was constructed for the rural only-child group and the rural non-only-child group; both models showed good fit (rural only-child group: χ²(6)=7.47, CFI = 0.997, TLI = 0.992, RMSEA = 0.019, SRMR = 0.019; rural non-only-child group: χ²(6)=6.74, CFI = 1.000, TLI = 0.999, RMSEA = 0.007, SRMR = 0.009).

In the rural only-child group, the R² for T2 depressive symptoms was 0.164 and for T2 internet addiction tendency was 0.250. The unstandardized coefficients (B), standard errors (SE), standardized coefficients (β), and 95% confidence intervals for the main paths were as follows: T1 internet addiction tendency positively predicted T2 depressive symptoms (B = 0.085, SE = 0.017, β=0.233, 95%CI[0.052, 0.118], p<0.001), but the prediction from T1 depressive symptoms to T2 internet addiction tendency was not significant (B = 0.176, SE = 0.118, β=0.064, 95%CI[−0.056, 0.408], p=0.137). In the rural non-only-child group, the R² for T2 depressive symptoms was 0.191 and for T2 internet addiction tendency was 0.260. The unstandardized coefficients (B), standard errors (SE), standardized coefficients (β), and 95% confidence intervals for the main paths were as follows: T1 internet addiction tendency positively predicted T2 depressive symptoms (B = 0.060, SE = 0.008, β=0.163, 95%CI[0.044, 0.076], p<0.001), and T1 depressive symptoms positively predicted T2 internet addiction tendency (B = 0.255, SE = 0.060, β=0.097, 95%CI[0.138, 0.373], p<0.001).

**Figure 4:**
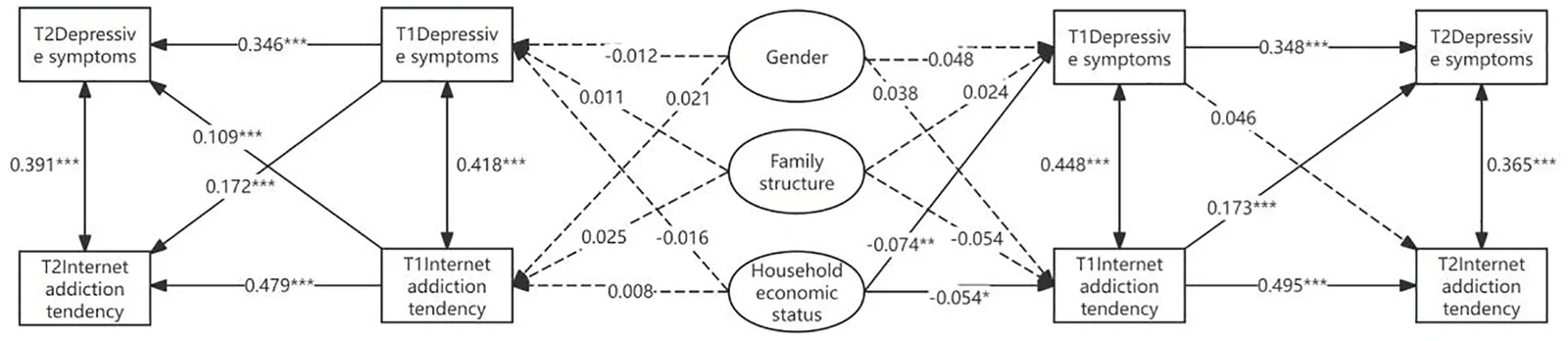
Cross-lagged model after cross-grouping. Note: The left side is the rural only-child group, and the right side is the rural non-only-child group. Solid lines in the path indicate p < 0.05, dashed lines indicate p > 0.05. Numbers on the lines represent standardized β coefficients. * p < 0.05, ** p < 0.01, *** p < 0.001.

In the cross-lagged paths, T1 tendency toward Internet addiction significantly predicted T2 depressive symptoms in both the only-child and non-only-child groups. However, T1 depressive symptoms predicting T2 tendency toward Internet addiction was significant only in the rural non-only-child group and not significant in the rural only-child group.The autoregressive paths for depressive symptoms and tendency toward Internet addiction were significant in both groups. See **Figure 4** for details.

#### 3.6.1 Between-group differences in cross-lagged paths

To examine whether the two cross-lagged paths”T1 internet addiction tendency → T2 depressive symptoms” and “T1 depressive symptoms → T2internet addiction tendency”differed significantly among the four groups, the Wald test was used for comparison. The results showed that the predictive coefficient of T1 internet addiction tendency on T2 depressive symptoms did not differ significantly among the four groups $\text{Wald }\chi^{2}\left(3\right)=2.66,\text{p}=0.448$, and the predictive coefficient of T1 depressive symptoms on T2 internet addiction tendency also did not differ significantly among the four groups $\text{Wald }\chi^{2}\left(3\right)=3.89,\text{p}=0.274$. These results indicate that although the significance of the “depressive symptoms → internet addiction tendency” path varied across groups in the subgroup analyses (significant in the urban only-child and rural non-only-child groups, non-significant in the urban non-only-child and rural only-child groups), the direct multi-group comparison (Wald test) showed that the path coefficients did not reach statistically significant differences among the four groups. Therefore, the current evidence is insufficient to determine a definitive moderating effect of urban–rural background and sibling status on the longitudinal relationship between depressive symptoms and internet addiction.

## 4 Discussion

This study conducted two follow-ups of 6, 070 first-year college students and used cross-lagged analysis to reveal a bidirectional relationship between depressive symptoms and tendencies toward internet addiction, while examining the moderating effects of urban–rural background and only-child status. The findings were: first, early tendency toward internet addiction predicted later depressive symptoms; this result held across all groups regardless of urban–rural background or only-child status. Second, the predictive effect of early depressive symptoms on later tendency toward internet addiction was significant only in urban only-child and rural non-only-child groups, but not in urban non-only-child or rural only-child groups, showing notable group differences. However, formal multi-group comparison (Wald test) showed that the path coefficients did not differ significantly among the four groups (Wald χ²(3)=3.89, p=0.274). Therefore, the current evidence is insufficient to confirm a definitive moderating effect of urban–rural background and sibling status on this longitudinal relationship; the subgroup differences in significance are more likely to reflect a weak trend that requires further verification rather than a confirmed group moderation mechanism.

### 4.1 The generality of internet addiction tendency predicting depressive symptoms

Regardless of urban–rural background and only-child status, T1 tendencies toward internet addiction significantly and positively predicted T2 depressive symptoms. This is consistent with existing literature reporting high comorbidity and longitudinal risk associations between internet addiction tendencies and depressive symptoms (6–11). This phenomenon suggests that, for freshmen just entering university, excessive internet use may serve as a universal early warning indicator associated with subsequent mental health problems. The formal multi-group comparison (Wald test) also demonstrated no significant difference in this path among the four groups, further indicating the cross-group consistency of internet addiction tendency as an early warning indicator for depressive symptoms.

### 4.2 Group specificity of the effect of depressive symptoms on tendency toward iInternet addiction

In contrast to the universality of the above path, the pathway from depressive symptoms to internet addiction tendency showed noteworthy group differences: T1 depressive symptoms significantly predicted T2 internet addiction tendency only in the urban only-child and rural non-only-child groups, whereas this path was not significant in the urban non-only-child and rural only-child groups. However, further multi-group Wald test indicated that the path coefficients did not differ significantly among the four groups (Wald χ²(3)=3.89, p=0.274), suggesting that this subgroup difference in significance is insufficient to infer a definitive group moderation effect and is more likely to reflect a noteworthy potential trend. We attempt to interpret this pattern of differences from the perspectives of family functioning, family support, and sibling relationships. It should be noted that these possible explanatory variables were not directly measured in this study; therefore, all the following theoretical interpretations are potential theoretical hypotheses of this study rather than empirical conclusions.

#### 4.2.1 Differences in family functioning and levels of family support

The McMaster Model of Family Functioning defines family functioning as the family’s ability to meet members’ basic physiological and emotional needs and to accomplish key developmental tasks, and includes six core dimensions: problem solving, communication, roles, affective responsiveness, affective involvement, and behavioral control (36).

Urban only-children and rural non-only-children represent two extremes of family support levels. Only-children, due to concentrated investment of family resources, typically enjoy greater parental attention and emotional support (15); rural non-only-children, however, face resource dilution and greater economic pressure, and may therefore receive less family emotional support (16). Previous studies can support this idea. Qi et al. found that family functioning can indirectly affect social media addiction through depressive symptoms, confirming the existence of the “family–emotion–addiction” mediation pathway (37). Mo PK et al.’s study of 5, 182 junior high school students showed from another angle that problematic internet use can indirectly increase the risk of adverse behaviors by weakening family support and exacerbating depressive symptoms through two pathways (38). In addition, the compensatory internet use theory (39) points out that when individuals face real-life emotional distress they are more likely to turn to the internet to seek escape; a lack of family support may amplify this transition. Cerniglia et al.’s research also indicates that poor family emotional responsiveness is closely related to youths’ tendencies toward internet addiction and psychological problems such as depressive symptoms and avoidance (40). Finally, Yu and Shek’s longitudinal study demonstrated from a developmental perspective that family functioning has a lasting predictive effect on adolescents’ internet addiction (41). Therefore, we propose a hypothesis: when individuals are situated at either the high end of family support (urban only-child) or the low end (rural non-only-child), depressive symptoms may be more likely to convert into internet addiction tendency. In the urban non-only-child and rural only-child groups, this conversion was not significant, possibly suggesting that their level of family support lies in the middle range or that other buffering factors exist; however, this inference still requires direct measurement of family functioning to be verified.

#### 4.2.2 The influence of sibling relationships

Several sibling relationships are essential for understanding internal differences in the non-only-child group. Kuang et al. proposed that sibling relationships are important mediators of relationships between family environment and adolescent psychological problems, helping individual experience support and buffering negative emotions (42). Gilligan et al.’s longitudinal research also confirmed that sibling warmth in non-only-child individuals can long-term decrease the risk that emotional problem will develop into problematic behavior (43). The protective role of sibling relationships may be affected by patterns of interaction in the family. Steffgen ST et al. found that when family resources are not abundant, parents’ conditional love and the perception of parental preference may easily cause competition and family conflict among siblings, weakening the protective effect of sibling support (44).

However, in our study, ‘depressive symptoms influencing tendency to internet addiction’ was not significant in the urban non-only child group, implying that the sibling relationship in this group may, on the whole, possess this expectation-benefiting buffering function;whereas the ‘depressive symptoms influencing tendency to internet addiction’ pathway was significant in the rural non-only child group, probably meaning that the sibling relationship in rural non-only child family is more damaged by resource competition and perceived parental favoritism, so that potential sibling support does not play a benefactive buffering role.Therefore, we propose a second theoretical hypothesis for the differences observed in this study: the non-significant path from depressive symptoms to internet addiction tendency in the urban non-only-child group suggests that their sibling relationships may have, on the whole, played the expected buffering role; whereas the significant path in the rural non-only-child group may indicate that sibling relationships in rural non-only-child families are more subject to interference from resource competition and perceived parental favoritism, preventing potential sibling support from forming an effective buffer. It should be noted that the above inference that sibling relationships among rural non-only children “may be poorer” is based on theoretical derivations from previous literature and is not directly supported by any data in this study. Verification of these hypotheses awaits future research that directly incorporates instruments such as sibling relationship quality scales and measures of perceived parental differential treatment.

#### 4.2.3 Advantages and limitations

Strengthening points of this study are: First, a relatively large sample size (6070 valid participants) was available, and attrition across the two surveys caused no significant structural bias on key variables (small effect sizes), thus ensuring the stability of the results. Second, compared with cross-sectional studies, the longitudinal design allows for a more reliable investigation of the temporal sequence and predictive direction among variables. Third, after controlling for covariates such as gender, family structure, and household economic status, we separately examined differences by urban–rural status and only-child status, and further cross-classified the two, providing new evidence on the interactive moderating effects of urban–rural and only-child identities.

The study also had the following limitations. First, the sample from a single university in Jingzhou limits regional and institutional representativeness. Second, the short follow-up with only two time points prevents observation of longer-term dynamics. Third, all main variables were measured by self-report, which may introduce shared method variance and overestimate correlations. Although the Harman test and CFA for PHQ-9 at T2 did not indicate extreme single-method effects, these tests cannot fully rule out common method bias. Self-report also limits self-awareness; future studies could incorporate multi-informant data (e.g., parent or peer reports). Fourth, despite controlling for gender, family structure, and household economic status, residual confounding from unmeasured variables (e.g., anxiety, loneliness, sleep problems, academic stress, social support, personality, and internet use duration/type) may still bias the estimated paths. Fifth, family support and sibling relationship quality were only theoretically posited as explanatory mechanisms but not directly measured, requiring further investigation. Sixth, the two-wave CLPM captures only group-level longitudinal prediction, not within-person causal inference. RI-CLPM would allow disentangling between- and within-person effects but requires at least three time points. Future research should collect three or more waves and adopt RI-CLPM to further verify the findings and examine subgroup differences.

## 5 Conclusion

Here we analyzed some longitudinal data on Chinese university freshmen based on cross-lagged models and revealed bidirectional longitudinal predictions between depressive symptoms and internet addiction tendency and the associated group differences. These conclusions reflect group-level predictions and should not be interpreted as within-person causal changes. Our conclusions are (1): The early tendency to addiction to Internet can distinctly forecast later symptoms of depression; this effect is confirmed by all four groups created based on the difference of urban and rural backgrounds and only or non-only children groups, which implies that the tendency to addiction to Internet is a longitudinal warning indicator to depressive symptoms for university students (2); In subgroup analyses, early depressive symptoms significantly predicted later internet addiction tendency only among urban only-child and rural non-only-child students; however, the Wald test showed no significant between-group difference in this path. Thus, current evidence cannot confirm that urban–rural background and sibling status moderate this relationship—the subgroup variation likely reflects a weak trend needing further verification. These findings support stratified mental health interventions: screening and preventing internet addiction tendency should serve as a universal strategy, while the subgroup analyses also suggest monitoring the potential risk of depressive symptoms converting to internet addiction among urban only-child and rural non-only-child students. Enhancing family support and sibling interaction remains a theoretical hypothesis awaiting empirical testing.

In summary, this large-sample longitudinal study revealed bidirectional associations and group specificity between depressive symptoms and internet addiction tendency. Future research should implement early screening of internet addiction tendency to identify depression risk, strengthen emotion regulation and family support for urban only-child and rural non-only-child groups, and deliver internet use interventions for urban non-only-child and rural only-child groups. Integrating school and family efforts and incorporating variables such as family functioning and sibling relationship quality can help develop precise stratified interventions. Subsequent studies should expand sample coverage, extend follow-up periods, and include parent and peer reports to reduce self-report bias. Family functioning, sibling relationship quality, and perceived parental emotional support can be examined as mediators or moderators to clarify mechanisms underlying group differences. In addition, potential confounding variables such as anxiety symptoms, loneliness, sleep problems, academic stress, perceived social support, personality characteristics, and the specific duration and type of internet use should be included in analyses, and more comprehensive control or instrumental variable methods should be used to reduce the interference of residual confounding on inference, thereby further clarifying the longitudinal relationship between depressive symptoms and internet addiction tendency.

## Data Availability

The raw data supporting the conclusions of this article will be made available by the authors, without undue reservation.
