# Supplementary material for: Cross-lagged analysis of depression symptoms and internet addiction tendencies among first-year university students and the moderating effects of urban–rural background and sibling status
Source: Front Psychiatry. 2026 Aug 26;17:1916116. doi: 10.3389/fpsyt.2026.1916116 (PMC13555227; doi:10.3389/fpsyt.2026.1916116)
Supplement: Supplementary file 1 [file Table1.docx]

## Medical Ethics Committee Research Project Approval Document

Document Code: KJ-LLSC(2.6.8.1) Version: 2024/A Applicable Department: Science & Education Section Project No.: 2024L0801

### Basic Project Information

Project Title: Longitudinal Observation on Mental Health Status of University Freshmen and a Controlled Study on Adjuvant Improvement of Mild Depression with ω-3 PUFAs Project Source: □ National □ Provincial □ Municipal □ Postgraduate Thesis □ Others Principal Investigator: Yu Ye Department: Non-Pharmacotherapy Department Discipline & Specialty: Psychiatry Research Direction: Non-Pharmacological Therapy Research Type: □ Basic Research ☑ Applied Research □ Comprehensive Research □ Others Research Funding: Postgraduate Thesis Project Funding Source: / Project Start Date: August 2024 Project End Date: April 2025

### Project Brief (Research Purpose & Significance)

This study aims to investigate factors affecting the mental health of freshmen in their first semester, and explore the efficacy of ω-3 PUFAs combined with CCBT for mild depression. Blood sampling will be conducted in advance to construct a low Ω-3 risk model, which will be matched with university student mental health questionnaires to analyze the impacts of lifestyle changes on college students’ mental health. A total of 55 blood samples will be collected from enrolled students of Yangtze University. Participants will complete questionnaires including PHQ-9, GAD-7, ISI, MSH27, and self-designed questionnaires covering general demographic information and lifestyle. Gas chromatography will be adopted to detect ω-3 PUFAs levels on the surface of blood cells. All collected data will be sorted and analyzed, and R language will be used to build a clinical prediction model for low ω-3 PUFAs status.

### Documents Submitted for Review

1. Research Integrity Commitment (V1.0)
2. Ethics Review Application Form (V1.0)
3. Research Staff Profile (V1.0)
4. Informed Consent Form (V1.0)
5. Research Protocol (V1.0)
6. Source Certificate of Biological Specimens & Information Data
7. Project Feasibility & Safety Demonstration (V1.0)
8. Conflict of Interest Statement (V1.0)
9. Recruitment Advertisement & Release Format (V1.0)
10. Statement on Forms of Research Outcome Dissemination (V1.0)

### Key Medical Ethics Review Checklist

1. Is the project application complete? □ Yes □ No
2. The research team has fully reviewed the Measures for the Administration of Drug Registration, Good Clinical Practice (GCP) and Guidelines for Ethical Review of Drug Clinical Trials, and will comply with relevant provisions. ☑ Yes □ No
3. The research protocol is scientifically designed and will be implemented in accordance with the design flow. ☑ Yes □ No
4. The trial carries low risks and can bring benefits to society, the hospital and participants. ☑ Yes □ No
5. Subjects will be recruited via standardized procedures, and all experimental risks will be fully informed. ☑ Yes □ No
6. A standardized informed consent form is developed; signatures will be obtained prior to participation. ☑ Yes □ No
7. Pre-trial laboratory examinations will be arranged to screen eligible subjects for this clinical trial. ☑ Yes □ No
8. Participants’ medical and legal rights will be fully protected. ☑ Yes □ No
9. All participants’ personal privacy will be strictly protected. ☑ Yes □ No
10. Does this research involve vulnerable populations? □ Yes ☑ No
11. Does this research involve patients with specific diseases or specific regional/ethnic groups? ☑ Yes □ No
12. Does this research involve genetic testing, storage and secondary utilization of biological specimens? ☑ Yes □ No

Issued by: Medical Ethics Committee of Jingzhou Mental Health Center

## Ethics Committee Review Record

Meeting Date: August 8, 2024 Venue: Administrative Building, Room 410 Attendance Summary: Total Required Members: ____; Actual Attendees: ____; Recused Members: ____; Absentees: ____

### Ethics Committee Member List

| **Name** | **Gender** | **Committee Position** | **Education** | **Title/Position** | **Specialty** | **Contact Number** | **Signature** |
| --- | --- | --- | --- | --- | --- | --- | --- |
| Tu Zheming | Male | Chairperson | Bachelor | Chief Physician / Party Secretary | Mental Health | 15926578889 |  |
| Nie Suocheng | Male | Vice Chairperson | Bachelor | Vice President | Mental Health | 13035302430 |  |
| Zhang Xinfeng | Male | Secretary | Bachelor | Attending Physician / Director of Science & Education Section | Mental Health | 13872293762 |  |
| Guan Peng | Male | Committee Member | Bachelor | Lawyer (Jingchu Law Firm) | Legal Service | 13797526655 |  |
| Li Mingxia | Female | Committee Member | Master | Lecturer, Deputy Director of Party Affairs Office, Yangtze University | Ethics Teaching | 18986660608 |  |
| Zou Mi | Female | Committee Member | Bachelor | / | Sociology | 13593816962 |  |
| Tao Yanqing | Female | Committee Member | Bachelor | Associate Chief Nurse / Director of Nursing Department | Psychiatric Nursing | 18064227766 | Tao Yan |
| Wu Youshan | Male | Committee Member | Bachelor | Chief Pharmacist / Director of Pharmacy Section | Pharmacy | 13507213523 |  |
| Chen Lan | Female | Committee Member | Bachelor | Deputy Section Chief of Discipline Inspection, Intermediate Librarian | Sociology | 13437208535 | Chen Lan |

### Voting & Review Outcome Table

| **Reviewed Materials** | **Approve** | **Approve with Revision** | **Re-review after Revision** | **Disapprove** | **Abstain** | **Suspend / Terminate Trial** | **Recusal** |
| --- | --- | --- | --- | --- | --- | --- | --- |
| Research Protocol |  |  |  |  |  |  |  |
| Informed Consent Form |  |  |  |  |  |  |  |

### General Review Opinion

Approve

### Follow-up Review Arrangement

Continuous Review Required: □ Yes □ No Continuous Review Frequency: □ 3 months ☑ 12 months □ 6 months Re-review Required: □ Yes □ No

### Final Approval Conclusion

☑ Approved □ Approve with necessary revisions □ Re-review after revision □ Disapprove □ Suspend / Terminate approved trial

### Supplementary Instructions

1. Strictly comply with GCP standards to guarantee the safety of all research subjects.
2. Promptly report all serious adverse events and protocol violations to the Ethics Committee.
3. Any revisions to the research protocol or informed consent form must be submitted to the Ethics Committee for filing or deliberation, and may only be implemented after obtaining official approval.

Signature of Ethics Committee Chairperson: ____________ Official Seal of Ethics Committee: ____________ Approval Date: ____________

### Contact Information of Ethics Committee

Full Name: Medical Ethics Committee of Jingzhou Mental Health Center Contact Person: Zhang Xinfeng Address: No.47 Taqiao Road, Shashi District, Jingzhou City, Hubei Province Postal Code: 434000 Contact Phone: 0716-8202157
